# The epistemic economy of vocal biomarker startups: a qualitative interview study of data practices and governance

**DOI:** 10.3389/fdgth.2026.1895383

**Published:** 2026-09-15

**Authors:** Abigail Yuhan, Sonal Swain, Robin Zhao, John Ramos, Anaïs Rameau

**Affiliations:** 1 Sean Parker Institute for the Voice, Department of Otolaryngology-Head and Neck Surgery, Weill Cornell Medical College, New York, NY, United States; 2 SUNY Downstate College of Medicine, Brooklyn, NY, United States

**Keywords:** digital biomarker, ethics, philosophy of science, qualitative study, startups, vocal biomarker, voice biomarker, epistemology

## Abstract

**Background and aims:**

Vocal biomarker technology has emerged as a promising non-invasive health assessment tool, with artificial intelligence enabling the screening, diagnosis, and monitoring of conditions including Parkinson's disease, Alzheimer's disease, depression, and heart failure. Vocal biomarkers are defined as vocal features, alone or in combination, validated as indicators of clinical outcomes. Startups in this space are generating substantial investment and anticipatory discourse around future commercialization, while the scientific foundation and governance framework remain unestablished. Drawing on Sabina Leonelli's epistemic economy framework, which analyzes how data are produced, valued, and governed within social, institutional, and commercial arrangements, we examine how voice data are converted into entities with clinical and commercial value, and at what scientific and ethical cost.

**Methods:**

We performed a qualitative interview study using an interpretive framework. Virtual semi-structured interviews were conducted with representatives from eleven vocal biomarker startups identified through purposive sampling. Interviews addressed data collection methods, IRB compliance, informed consent, participant demographics, geographic sourcing, and financial incentives. Two researchers independently performed thematic analysis, reconciling differences via discussion.

**Results:**

Six themes were identified: (1) the absence of shared data standards introduced substantial hardware and protocol heterogeneity, uncontrolled confounding, and label noise, undermining scientific validity; (2) IRB engagement ranged from robust multi-site review to complete absence of oversight; (3) consent practices varied from granular opt-in to implied consent and minimal disclosures; (4) voice data were acknowledged as inherently non-anonymizable, creating unresolved privacy and re-identification risks; (5) data sourcing was asymmetric, with collection from low- and middle-income countries in 4 out of 11 startups while targeting commercial deployment in high-income countries; and (6) commercial incentives produced proprietary protocols that preclude independent validation and impede data sharing.

**Conclusion:**

The vocal biomarker startup ecosystem operates as an epistemic economy in which methodological, regulatory, and governance choices tend to favor commercial interests and high-income populations over scientific rigor and equity. Standardized data provenance requirements, reassessment of voice data's risk classification under existing privacy frameworks (e.g., HIPAA, GDPR), and participatory governance including source communities are urgently needed to ensure vocal biomarker products are scientifically valid, ethically grounded, and equitably developed.

## Introduction

The use of artificial intelligence (AI) in healthcare has created novel opportunities to learn from large human datasets (1, 2). Among these signals, voice is emerging as a richly encoded physiological marker that can be collected non-invasively for disease detection and monitoring with ubiquitous smart phone technology (3, 4). The increasing commercial interest in the space is well demonstrated with the multiplying numbers of health startups applying AI to voice (5). Recently, one of these startups, Canary Speech, has collaborated with Microsoft to integrate vocal biomarker technology into their ambient scribe technology, Dragon Copilot, for the early detection and management of mental health and cognitive conditions, marking one of the first implementations of vocal biomarker technology into clinical workflows (6).

Despite the commercial enthusiasm and recent clinical application of vocal biomarker technology, the field seems to be limited by a fundamental challenge: the limited availability of well annotated voice data collected with a sound acquisition protocol. The Bridge2AI Voice Consortium is working on tackling this issue with a multi-million dollar support from the National Institute of Health (NIH) Common Fund. However, the lack of Food and Drug Administration (FDA) approval to date and limited adoption of the AI tools has created pressures on vocal biomarker startups, with pressing investor demands for return on investment. One bottleneck limiting startups' technological advancement is the challenge of voice data collection, in large part related to data privacy concerns and regulations. Voiceprints are among the eighteen identifiers enumerated under the HIPAA. Privacy Rule's Safe Harbor standard (7). Under the European Union's General Data Protection Regulation (GDPR), voice recordings constitute biometric data subject to special-category protections when processed for the purpose of uniquely identifying a person (8).

When training data are extracted from certain communities and used to benefit others, the idea of epistemic fairness—the symmetric creation, distribution, and recognition of knowledge—is put into question. Nambiar et al. discusses how colonial legacies, disparities in funding, and extractive partnerships shape knowledge production by the Global North, who often dictates data access and use (9). Tossas-Milligan and Winn articulate that those from higher-resourced environments are often those determining innovating and developing precision medicine, which leads to bias towards these high-income environments (10). This bias leads to a cycle of health inequity, perpetuated by these highly resourced institutions.

These concerns about fairness can be situated within a broader descriptive frame: the “epistemic economy” that Sabina Leonelli develops in her paper, “How Data Cross Borders: Globalising Plant Knowledge through Transnational Data Management and Its Epistemic Economy” (11). Leonelli introduces an “epistemic economy” to describe a knowledge system that encompasses a particular vision for research, the technical tools through which that vision is realized, and its social and economic motivations. She discusses the epistemic economy of plant data infrastructures, which present “global plant knowledge production as a common good to be harnessed for the continuing survival of our species and of the planet,” while standing in “complex relationship with attempts to control or sanction access to plant data resources in ways that reflect national and/or private interests.” Leonelli's framework thus provides a lens for analyzing startups' voice data collection practices and for understanding how these data are produced and governed within a complex social and ethical landscape.

Previous research from the Bridge2AI Voice Consortium has mapped the landscape of vocal biomarker startups, analyzed language on their websites, and assessed regulatory practices in this space (12, 13). Findings revealed a significant amount of hype fueling startups around vocal biomarker technology and variable transparency around data collection practices based on website information. Ethicists in the consortium have called for transparency in the methods to ensure standard methods and regulations were observed (12). However, more investigation is needed into the practices of the startups through firsthand accounts to clarify how data collection is performed, what ethical frameworks govern data collection and what scientific standards are in place. We have focused deliberately on startups exclusively. Although many of the challenges of voice data (e.g., its non-anonymizability, the absence of shared standard) confront academic researchers as well, startups occupy a distinctive position in the ecosystem. They operate under investor timelines and commercialization pressure, largely outside the institutional infrastructure of mandatory ethics review, funder data-sharing mandates, and research-integrity training that constrains academic research, and they are increasingly where the field's data accumulates. Data collection practices in academic voice research have been characterized separately through a national survey (21). Our study aimed (1) to fill this gap in knowledge through a qualitative interview study with vocal biomarker startups and (2) to provide an epistemic and ethical analysis on current practices of vocal biomarker startups. Specifically, we sought to characterize the methodologies and governance frameworks that startups themselves apply to voice data, and to identify gaps in these practices that future research and regulation could address.

## Materials and methods

### Study design

In this study, we used an interpretive qualitative framework to investigate vocal biomarker startups' data collection practices, the ethical frameworks governing data collection and the scientific methods and standards currently in place. This study is reported in accordance with the Consolidated Criteria for Reporting Qualitative Research (COREQ); the completed checklist is provided as Supplementary Material. Semi-structured interviews were performed. The study was deemed exempt by the Weill Cornell Medical College Institutional Review Board (IRB) (#24-02027010-02).

### Study sample

Vocal biomarker startup representatives were recruited by email via purposive sampling from the startup registry maintained by the Bridge2AI-Voice Consortium, which builds on the consortium's published systematic mapping of the vocal biomarker startup landscape, which identified 27 active companies identified worldwide as of July 2023, with their locations, sizes, funding, and products (5). This list is continuously updated through the stakeholder networking efforts culminating in the yearly Voice AI Symposium (14). Twenty-seven companies were approached, of which eleven participated (41%); 16 did not respond to two contact attempts. Purposive selection aimed at diversity of geography, company maturity, and clinical focus rather than proportional representation of the field. Company characteristics (headquarters location, founding period, and funding stage) were compiled from the interviews supplemented by publicly available company information. Two clinical researchers (RZ, male, and SS, female) and the senior female author (AR), a laryngologist expert in vocal biomarkers, conducted interviews with 11 English-speaking representatives from vocal biomarker AI companies worldwide, with sample size determined by thematic saturation and adequate representation of startup geographic diversity and maturity.

### Data collection

Participants received a brief introduction to the research study before the interview, including rationale for conducting the research and understanding startups data collection procedures, governance frameworks scientific standards. Following the brief introduction, participants engaged in a one-on-one semi-structured interview with one or two members of the research team (RZ, SS and AR). The interviews were not initially pilot tested and were not repeated. Participants were provided with open-ended questions with minimal feedback from the interviewer. Interviews were conducted via Zoom, and recordings were transcribed verbatim using a combination of Zoom's built-in transcription (using a HIPAA-compliant Zoom platform) (15). Due to the virtual nature of the interviews, no field notes could be gathered. The interview questions (Appendix 1) focused on the identification of target research participant populations, consent procedures, data collection methods, financial incentives, demographic and geographic information on participant populations, and guiding ethical principles. All eleven transcriptions were reviewed for accuracy by the individual who conducted the interview and were not returned to the participants for review. Interviews were completed within 30 min. The interviews were conducted between February 2025 and May 2025.

### Data analysis

The qualitative data obtained from these interviews were coded and analyzed using MaxQDA software by VERBI (version 24) to identify common themes (16). The first two interviews performed were inductively coded via thematic content analysis by the clinical researchers (RZ and SS) with independent generation of codebooks by each author. Codebooks were reconciled via consensus discussion with the generation of a finalized codebook that was used to deductively code all 11 interviews. The coding for the rest of the interviews was performed independently by the same 2 researchers, and coding differences were reconciled by consensus discussion with the senior researcher (AR). Study researchers included a first year medical student (SS), a postgraduate clinical researcher (RZ), and an academic laryngologist (AR). Thematic saturation was reached. The analysis was not shared with the startups for feedback.

### Reflexivity statement

Members of the research team, including the senior author, are investigators in the Bridge2AI-Voice Consortium, an NIH-funded effort to build standardized, ethically sourced voice datasets, that is, an initiative that aspires to shape norms in the very field this study examines. This position afforded access to startup representatives and domain expertise in vocal biomarker research, but it also means the team approached the interviews with prior commitments to data standardization, research ethics oversight, and open science that may have shaped both what representatives chose to disclose and how their accounts were interpreted. We sought to mitigate these influences through dual independent coding with consensus reconciliation, the use of verbatim quotations linked to anonymized company identifiers, and the reporting of per-company counts for factual characteristics so that readers can audit the evidentiary basis of each claim. We acknowledge that recruitment through the consortium's network likely selected for companies already engaged with the academic and ethics community.

## Results

### Participant characteristics

A total of eleven individual startup representatives interviews were conducted. Startup characteristics are presented in Table 1. Three of the startups were based in the United States, two in Canada, one in the United Kingdom, one in Luxembourg, one in Germany, one in France, one in Australia, and one in Argentina. Three of the startups were started in the last five years, four in the last ten years, one in the last 15 years, two over 20 years ago, and one had an unclear start date. One startup was in a seed stage, two were in series A stage, two were in series B stage, one was in series C stage. Two startups were funded through government grants, one was publicly traded, one was a nonprofit, and one was bootstrapped. Most startups applied AI to a specific clinical purpose along the care pathway, whether screening, early detection, diagnosis, or longitudinal monitoring, rather than to disease management broadly. The conditions most frequently targeted were Alzheimer's disease, type 2 diabetes, and depression. Only one startup discussed extending the field to non-English speakers, particularly Spanish-speaking populations.

**Table 1:** Startup characteristics.

ID	HQ country	Clinical target(s)	Data collection countries (LMIC?)	IRB/ethics status	Consent format	Compensation	Data sharing
A	USA	Respiratory (asthma); COVID-era study; multi-condition ambitions	US; India (∼50,000); Europe (<1,000); 4 African countries (∼10,000). LMIC: YES	Both: academic partner IRBs + Western IRB (commercial central)	Mixed: e-consent default; waiver of consent sometimes; occasional partner paper consent	Yes: US min $25, up to few hundred $ longitudinal;	Shares via DUAs/mutual license agreements; no-cost collaborations
B	France	Psychiatry	France only. LMIC: NO	French ethics committee approval	Robust written (leaflet + tick-box; deliberately declined legal implied-consent route)	No (illegal in France)	Not discussed
C	United Kingdom	Mild cognitive impairment	UK, US. LMIC: NO	UK standard ethics review + US commercial central IRBs	Granular opt-in (item-by-item checkboxes)	Yes (cash, standard rate, capped)	Mixed: some data was released, some is proprietary
D	Argentina	Parkinson's, Alzheimer's, cognitive screening	Argentina, Chile, Peru, Colombia, Mexico, Brazil, US, N. Ireland, Spain, Kenya, Egypt, Ethiopia, Philippines. LMIC: YES	None sought by company (internal ethics committee for consent; partners use own committees)	Granular online opt-in + digital signature; paper in Kenya (partner-managed)	No (meals + transport only)	Proprietary
E	Canada	Alzheimer's/CNS dementia, psychiatric, hepatic encephalopathy, oncology/immunology cognition, Huntington's, Parkinson's	US; Canada; Australia; clinical trials “globally”. LMIC: NO	Commercial third-party ethics board	Mixed: own REB-approved written forms; delegated to sponsor for clinical trials	Yes (∼$20/hr)	Mostly owned by pharmaceutical companies the startup works with
F	USA	COVID-19, pneumonia, respiratory; dementia populations	Colombia, India, Pakistan, US, Japan. LMIC: YES	Ethics approval obtained in some countries and not others (Colombia)	Written form via nurse; verbal assent; proxy consent for dementia; granularity unclear	No (“wasn’t allowed in… India”)	Unclear (hospital agreements; describes negative incentives to share)
G	Australia	Parkinson's, NF1, Friedreich's ataxia, AT, MS, dementias/aphasia, pediatric neurodev.	320 sites worldwide “except Africa and Antarctica”; mostly Europe/N. America; biggest in Australia. LMIC: YES	Institutional/academic (university; interviewee is chair professor)	Mixed: patient-led simple assent to formal signed consent + plain-language statements	No (“the joy of participating”)	Services pharmaceutical companies that owned the voice data
H	Luxembourg (academic project + spinoff)	Diabetes, mental health, fatigue	Remote, “from all over the world”; French/US/UK named. LMIC: NO	Institutional/academic ethics process	Robust written signed; concise-vs-complete compromise with DPO	No	No
I	Canada	Diabetes (primary); hypertension	India (multi-site, via CRO); Canada; US designed; Africa + S. America in progress. LMIC: YES	IRB always obtained for every study	Robust written (standard informed consent doc, walked through, signed)	Mixed: most studies yes; “India… a few dollars a day. In the U.S… a lot more”	Proprietary (“that's proprietary… industry research lab”)
J	USA	Mental health (e.g., depression) in occupational screening	Greece, US; Philippines upcoming. LMIC: NO	None sought (planned via future university collaboration; “So what's IRB?”)	None	No (mandatory part of occupational health screening)	Mixed: free anonymized data to research orgs; no commercial licensing
K	Germany	Mental health: ADHD, dementia, depression	Mostly Germany; Europe; future English/Spanish-speaking countries. LMIC: NO	Institutional via clinical partners’ own ethics procedures	Mixed: partner in-clinic paper + digital consent	Mixed: patients mostly no; healthy controls yes	Proprietary (“not making them public”)

### Overall qualitative results and themes

Six overarching themes were identified from the consensus coding: (1) variability in audio capture and labeling as a risk to scientific validity; (2) heterogeneity in IRB engagement and regulatory oversight; (3) unresolved consent challenges for vulnerable populations; (4) non-anonymizability and re-identification risk; (5) Global data sourcing, generalizability, and epistemic exploitation; (6) Commercial incentives, data stewardship, and barriers to validation.

#### Theme 1: variability in audio capture and labeling as a risk to scientific validity

Exemplar quotes regarding variability of data collection protocols are shown in Table 2. Startup representatives described difficulties arising from heterogeneity in data collection, noting that “different phones [have] different recording frequencies” and “recording the voice on a laptop vs. another laptop vs. a phone has big differences” (Q2–Q3). This variability in sampling rates and capture hardware introduces measurement noise that threatens the reliability, and ultimately the validity, of this type of research.

**Table 2:** Exemplar quotes: variability in audio capture methods and Its uncertain impact on scientific validity.

Theme	Exemplar quotations
Recording heterogeneity and hardware inconsistency	Q1 (startup C): “And it turns out that at the sort of level of how the Facebook app interacts with the phone hardware, it is not able to turn on the microphone. But no one is going to tell you this. And basically if you run an app that works perfectly well in a standard browser and you try and run it there, you just don't get the audio, but it's kind of hard to troubleshoot. And there's like a thousand small engineering things like this related to collecting audio in the browser.” Q2 (startup F): “Different phones have different recording frequencies… lossy compression is a big problem…” Q3 (startup I): “Recording the voice on a laptop vs. another laptop vs. a phone has big differences.” Q4 (startup G): “One Samsung phone vs. another… different microphones… affects sampling rate and sensitivity…” Q5 (startup K): “Different kinds of encoding of audios from different devices… again can be correlated with the data.” Q6 (startup H): “Anyone can participate with any device… we have a lot of heterogeneity, but that's one limit and power of the study.”
Confounding variables and label quality	Q7 (startup A): “It's very easy to be misled in thinking you have a vocal biomarker or speech feature that is obviously different between your positive and negative cohorts. So it's very difficult to make absolutely sure… what kind of possible factors are impacting what you're seeing.” Q8 (startup F): “The label is important because there's this thing called label noise… let's say we're collecting data from asthma patients but then one doctor says it's asthma but the other says it's not—that can totally destroy your algorithm.” Q9 (startup K): “There's a high chance that these two groups will differ in more than just the condition… you have to deal with great care of matching the controls to the experimental group as you cannot randomize them.”
Reproducibility and standardization gaps	Q10 (startup C): “I would love for it to just be a standard that audio data is collected even in studies that are not particularly about audio data collection. And obviously this needs to be an opt-in for the participant.” Q11 (startup I): “I wish there was a lot of public datasets that we could play with… but it's quite difficult… to create datasets of voice that are very… difficult to study.” Q12 (startup F): “There's negative incentives for sharing data… it's not fully anonymous… finding ways to break those barriers…” Q13 (startup H): “We need to have more ethical and standardized guidelines as to how to collect data, how to process the data, how to share it.”

The most damaging form of this confounding effect occurs when a confounding variable coincides exactly with the outcome. If the disease cohort is recorded in clinical settings and the controls at home, device and environment differences align perfectly with the disease label, and the model can separate the groups on recording context alone without learning anything about the disease. As one interviewee noted, “there's a high chance that these two groups will differ in more than just the condition… you have to deal with great care of matching the controls to the experimental group as you cannot randomize them” (Q9).

Label noise is an additional problem, exemplified by one interviewee stating, “The label is important because there's this thing called label noise… let's say we're collecting data from asthma patients but then one doctor says it's asthma but the other says it's not—that can totally destroy your algorithm.” (Q8). Whereas confounders create systematic differences in the inputs, label noise affects the reliability of the outcome label itself. If physicians disagree on whether a patient has hypertension, the ground-truth label used to train the algorithm is itself uncertain, and a model trained on inconsistent labels inherits that ambiguity (17–20).

Together, these issues of methodological heterogeneity, confounders, and labeling consistency reflect the challenges with reproducible vocal biomarker research. The startups reported no shared standards for collecting voice samples. Vocal biomarker data are therefore being accumulated at scale without norms, with each organization developing its own protocols that cannot readily be reconciled with those of others (21). The comparison with blood banking is instructive: blood banking matured within an established framework of standards for collection, storage, provenance documentation, and ethical governance (22), whereas voice data have no equivalent. Validation of AI voice models depends on cross-validation, replication, testing generalizability to new datasets, and ensuring the datasets are not biased (23), yet none of these is achievable across organizations without shared metadata. As a result, findings from one study cannot be meaningfully compared with or pooled across others in the field, creating significant epistemic challenges.

#### Theme 2: heterogeneity in IRB engagement and regulatory oversight

Exemplar quotes regarding IRB issues, regulatory compliance, and privacy concerns are shown in Table 3. Ethical oversight varied widely across the startups, ranging from robust IRB processes to serious gaps. At one end of this spectrum, organizations with established academic partnerships or university affiliations described systematic IRB submission and multi-site review. At the other end, two of the eleven startups had sought no ethics review of their own: one, a business whose founder was unfamiliar with the IRB process entirely, planned review only through a future university collaboration (Q18); the other reasoned that its team members at academic institutions “do not require that information” (Q19). Among the nine that did obtain review, three used commercial or central IRBs, which may itself warrant scrutiny given that such entities offer expedited approval. A further organization described itself as “purely private” with “no connection with any public institution or university” (Q17), yet had in fact obtained approval from a national research ethics committee for its clinical study, suggesting that startups may position themselves outside the research-ethics system even when they are formally within it. This range suggests that the vocal biomarker startup ecosystem currently operates without a consistent baseline of ethical oversight, and that the regulatory architecture governing human subjects research has not kept pace with the commercialization of vocal biomarker.

**Table 3:** Exemplar quotes: IRB, regulatory compliance, and research laxity.

Theme	Exemplar quotes
Commercial IRB gaps	Q14 (startup E): “We used a third-party ethics board submission because we're not associated with a university…” Q15 (startup C): “We are a company. We are based primarily in London, in the UK. So we did submit to IRB and we went through the standard of IRBs here in the UK. And then for the data collection that we've done in the US, we've used commercial IRBs, specifically central IRBs. We can get an approval and then you can use it across sites.” Q16 (startup C): “And I think most of the people who are collecting voice data are also some commercial entities. But especially you know if you're sort of running an academic lab, [they] don't have the same levels of rigor.”
No IRB submitted	Q17 (startup B): “Our project was purely private. We had no connection with any public institution or university, etc.” Q18 (startup J): “No, we were a business. So actually, we were going to have an IRB with a collaboration with a university. We don't anticipate any issues, but I'm having GDPR kind of like constant in that case.” Q19 (startup D): “So it's not me, so I don't work for an academic institution. We have some people on the team working for academic institutions, but they do not require that information, so we don't do it.”

#### Theme 3: unresolved consent challenges for vulnerable populations

Exemplar quotes regarding ethical and consent concerns are shown in Table 4. Consent emerged as a theme with two distinct dimensions: the procedural mechanics of obtaining consent, and the deeper ethical question of whether consent is meaningful when participants may not fully understand what they are consenting to or when they lack capacity to consent at all.

**Table 4:** Exemplar quotes: unresolved ethical questions around consent for vulnerable populations.

Theme	Exemplar quotes
Minimal consent format	Q20 (startup A): “Because our studies have been… non-significant risk studies. Sometimes we even get a waiver of consent.” Q21 (startup B): “Apparently, we could have just allowed the patient to start the study, and by doing so, it would implicitly mean they did not oppose participation.” Q22 (startup J): “I'm just having parts of the GDPR—that's how we use the data, what we're going to do, how we're processing them—and they give us consent. Any questions or withdrawal will just come back to us and we delete it. That's it. It's kind of like a one-page format, that's it.”
Consent delegated to sponsors	Q23 (startup E): “For the clinical trial studies, the consent process is assumed by the pharmaceutical company or the sponsor of the trial. And so we have no actual… we're not approving their consent language, but it is up to them to make sure it's included.”
Consent readability tension	Q24 (startup H): “We wanted to make it short, and the data protection officer wanted to make everything long… we tried to make a compromise between explaining well to participants but also having it concise.”
More robust consent as a positive example	Q25 (startup C): “We have a standard basic consent where they're taking individual boxes of what they're consenting to.” Q26 (startup G): “They have to sign… read plain language statements… terms of use… can withdraw anytime.” Q27 (startup D): “We have an informed consent form… they sign via a digital signing platform… in Kenya… they sign actual paper.”

Consent practices varied widely across the startups. At one end, one organization reported that its studies received waivers of consent on the grounds of being “non-significant risk,” a categorization that warrants scrutiny given the biometric properties of voice data and the risk of re-identification. Others described more robust, granular consent procedures, indicating that strong practice is achievable within the same field.

Beyond these procedural differences, the interviews raised questions of the ethical soundness of the consent processes itself. One startup revealed a potentially concerning attitude toward consent, explaining: “We wanted to make it short, and the data protection officer wanted to make everything long… we tried to make a compromise between explaining well to participants but also having it concise” (Q24). Prioritizing brevity in this way risks sacrificing participants' understanding of what they are agreeing to, which is the substance of meaningful consent rather than a matter of form.

Guidance from the Institute of Medicine (US) Committee on Assessing the System for Protecting Human Research Participants frames consent as an informed, interactive, and ongoing process rather than a one-time formality, a standard that a deliberately shortened disclosure may fail to meet (24).

#### Theme 4: non-anonymizability and re-identification risk

Exemplar quotes regarding the inability to anonymize data and the resulting privacy risks are shown in Table 5. Voice is an unusual piece of biomedical data: it may serve as a clinically meaningful biomarker while also functioning as a highly identifying piece of information (25). This dual character is the crux of the problem, because the same signal that carries diagnostic value also carries identity, and the two cannot be separated without destroying the data. Interviewees spoke to both aspects, and in doing so exposed a gap in privacy regulation.

**Table 5:** Exemplar quotes: Non-anonymizability and privacy risk.

Theme	Exemplar quotes
Voice as inherently identifiable data	Q28 (startup E): “Voice data in and of itself is personally identifiable… instruct our transcriptionists to redact… we have training once or twice a year.” Q29 (startup F): “Full anonymization might not be possible because the sample is a voice.” Q30 (startup I): “The de-identification of voice is very problematic… I can’t change the voice because… then it's not useful.”
Data breach severity	Q31 (startup F): “If your database of voice samples is hacked, that could be a severity incident because it's hard to anonymize—or maybe it's impossible.” Q32 (startup F): “There's negative incentives for sharing data… if Stanford accidentally leaks the data, then I'm kind of partially responsible.”
Security through access restriction	Q33 (startup B): “There was only one person in the company able technically to access the raw data, who was the data scientist…” Q34 (startup C): “We have very secure infrastructure, compliant infrastructure… it's a lot of work to set up and to maintain.”

Representatives described voice as inherently identifiable and pointed to the difficulty of stripping identity from it: “The de-identification of voice is very problematic… I can't change the voice because… then it's not useful” (Q30). Another organization echoed this concern, noting that a breach of a voice database would be especially severe precisely because the data are intrinsically tied to individual identity. Because these data are largely collected and held by commercial entities, this irreducible privacy risk shapes a knowledge environment in which the incentives work against data sharing rather than toward it.

#### Theme 5: global data sourcing, generalizability, and epistemic exploitation

Exemplar quotes regarding the use of global data and the resulting concerns about exploitative data sourcing are shown in Table 6. Four of the eleven startups collected data from low- and middle-income countries (LMICs), three of them in India. Startups noted that compensation in these settings is lower than it would be in high-income countries (HICs): “In India, it's a few dollars a day. In the U.S., it would be a lot more” (Q41). Yet while these companies sourced data in LMICs, their products were intended primarily for HIC markets (Q39–Q40). For one startup the asymmetry was also quantitative: it reported approximately 50,000 research participants in India and 10,000 across four African countries, against fewer than 1,000 in Europe. By the startups' own account, the lower compensation standards in LMICs reflect a structural incentive to collect data wherever it is cheapest. This pattern, extracting biological data from countries with weaker regulatory protection and commercializing the resulting products in high-income markets, exemplifies the asymmetry at the heart of what Leonelli terms the epistemic economy of transnational data: the channeling of data collected from communities in the Global South into systems that are controlled by, and primarily benefit, powerful players in the Global North (11). Leonelli describes this same dynamic in agricultural data as a form of digital bioprospecting (11).

**Table 6:** Exemplar quotes: global data sourcing patterns, generalizability concerns, and risks of epistemic exploitation.

Theme	Exemplar quotes
Data collected from LMIC populations	Q35 (startup I): “That was in India… we collected the data using a CRO in India.” Q36 (startup A): “We've done quite a few in India… one collaboration in four African countries.” Q37 (startup F): “In Colombia, India, Pakistan, US, Japan.” Q38 (startup D): “We work with indigenous people in Peru, we work with university populations in Latin America and the United States, we work with a third age in hospice populations in Spain.”
Products primarily for HIC market	Q39 (startup I): “Canada will be first.” Q40 (startup I): “We have an app… it's been registered as a Class One medical device in Canada.”
Compensation inequities in LMICs	Q41 (startup I): “Most of them, yes… in India, it's a few dollars a day. In the U.S., it would be a lot more.” Q42 (startup F): “We didn't [compensate]. I think because it wasn't allowed in at least in India… some ethical codes.”
Demographic homogeneity and non-English language gaps	Q43 (startup E): “It was very, very difficult to get non-English data.” Q44 (startup E): “We've had some success with MTurk with older adults… looking for multi-language participants… very, very difficult to get non-English data.”
Absent or limited patient advocacy engagement	Q45 (startup C): “I think it can be helpful to ask patients or you know by proxy advocacy groups what they actually care about… and then maybe the final thing that I would love to see more of is giving back to the people who contribute their data.” Q46 (startup K): “The aim is to involve them at every stage of the process… they carry a very particular perspective that might be informative.” Q47 (startup H): “It's very important to have them on board. From the beginning, they have to understand what vocal biomarkers are and what they could gain from it. Because if they are not on board, then nobody will use the technology.”

A further gap concerned the limited engagement of patient advocacy groups in study design (Q45–47). Notably, the case for their involvement came from the startup representatives themselves, who described advocacy groups as a source of perspective that the research would otherwise lack. One noted the value of asking “patients or you know by proxy advocacy groups what they actually care about,” adding that they “would love to see more of… giving back to the people who contribute their data” (Q45). Another framed involvement as continuous rather than consultative, aiming “to involve them at every stage of the process” because “they carry a very particular perspective that might be informative” (Q46). A third stressed early engagement: “From the beginning, they have to understand what vocal biomarkers are and what they could gain from it. Because if they are not on board, then nobody will use the technology” (Q47). These aspirations were realized unevenly. Four startups described structured engagement with patients or advocacy groups in their research, four described engagements as aspirational only, and three reported none. That a majority had no structured engagement despite recognizing its value highlights the gap between endorsing participatory input and building it into study design. It also reinforces the asymmetry described above, i.e., the populations whose voices are collected remain largely without a structured role in shaping the research.

#### Theme 6: commercial incentives, data stewardship, and barriers to validation

Exemplar quotes regarding concerns about the commercialization of voice data into products are shown in Table 7. Seven of the eleven startups had developed commercial products from their research datasets and three more were in the process of doing so. The data collected feed into commercial products, giving these organizations a financial incentive both to collect data and to protect them as corporate assets (26). This tension between scientific progress and commercial control surfaced repeatedly. Several startups expressed a preference for keeping data proprietary, as one explained: “There's negative incentives for sharing data… it's not fully anonymous… if you share your data to [another institution] and then they accidentally leak the data, then I'm kind of partially responsible… people are not incentivized to share it because then it's like their research will be overtaken” (Q49). This quote captures how a legitimate concern (the non-anonymizability discussed above) and a competitive concern (being “overtaken”) are entangled: privacy risk supplies a defensible rationale for withholding data, while financial and reputational interests supply the underlying motive.

**Table 7:** Exemplar quotes: commercialization tension and data stewardship.

Theme	Exemplar quote
Proprietary data and negative incentives for sharing	Q48 (startup I): “A lot of our data is not publicly available because we're an industry research lab, so it's proprietary.” Q49 (startup F): “There's negative incentives for sharing data… it's not fully anonymous… if you share your data to [another institution] and then they accidentally leak the data, then I'm kind of partially responsible… people are not incentivized to share it because then it's like their research will be overtaken.”
Open data as a counter-model	Q50 (startup C): “All of this data that's collected…is being made openly available online through the…platform.” Q51 (startup A): “We've also done some no-cost collaborations… just like a data use agreement.”
Commercial product development from research datasets	Q52 (startup A): “Yes, we've definitely developed multiple products from our research data sets.” Q53 (startup D): “We have 32 clients spanning through Latin America, US, Europe, Africa, and Oceania.” Q54 (startup C): “We have a product now… which is a test for mild cognitive impairment that anyone can do in any browser on any device, take 10 min to do.”

This proprietary control has direct consequences for the reliability as a whole. When each organization trains and validates its algorithms on its own closed dataset, no independent mechanism exists to verify whether reported performance reflects a genuine biological signal or merely idiosyncratic features of that dataset. Validity claims therefore cannot be checked across organizations, which is precisely the reproducibility problem identified in theme 1, now reinforced by commercial structure rather than only by methodological heterogeneity.

A small number of counter-models emerged within our sample that suggest alternatives to this proprietary logic are possible. Open data releases through platforms and no-cost data use agreements between collaborating organizations suggest that some startups in the field are attempting to build more permeable and sustainable data infrastructure. One mechanism for reconciling these competing pressures is a data trust. Chan et al. outlines such a trust, which would pool training data, establish a verification regime to verify that model developers only use the trust's data, and offer incentives for developers to submit to the regime (27). A neutral custodial institution of this kind can redistribute control over data from private holders toward a shared resource with the public. More broadly, open data frameworks offer a route to address structural inequalities, by incorporating stakeholders feedback, allowing data collection to be contextualized, and keeping data available, even when individual organizations falter (28).

## Discussion

The findings of this study reveal social, ethical, and legal concerns within the vocal biomarker startup ecosystem. While these companies claim they are doing innovative work that can improve human health outcomes, they operate under significant time pressures and short investment timelines. This environment may lead to compromised methodological practices, including questionable decision-making such as the collection of sensitive voice data in other countries where regulatory oversight may be less stringent. Our interviews revealed that the methodological and governance choices made by vocal biomarker startups are not isolated technical decisions, but mutually reinforcing choices shaped by commercial incentives. Two other studies of vocal biomarker startups, using different methods, reached compatible conclusions. A discourse analysis of startup websites found that most companies relied on promissory language, emphasizing accuracy and future impact, while disclosing little about their training data; the majority provided no description of these datasets at all (12). A scoping review of the field's ethical, legal, and social implications similarly documented a concentration of research and industry in the Global North and noted that companies often treat compliance with GDPR and HIPAA as sufficient governance, despite these frameworks not being designed for the specific risks of voice data. Privacy regulation can also function rhetorically: in our interviews, the risk of re-identification associated with voice data was invoked to justify withholding datasets (Q49). The same regulatory framing that protects participants can thus also reinforce limited data sharing, which in turn constrains independent validation, transparency and progress in the vocal biomarker field. That three studies using different methods arrive at compatible conclusions suggests that the same patterns likely characterize the field, not just individual companies.

Several of the challenges documented here are not unique to startups, and situating our findings within the broader vocal biomarker field clarifies what is and is not distinctive about the startup ecosystem. The non-anonymizability of voice (theme 4) is an intrinsic property of the data that confronts any collector, academic or commercial (25). The absence of shared data standards (theme 1) is likewise a failure in the field generally: a national survey of academic voice researchers documented similar protocol heterogeneity and barriers to data sharing in university settings (21), and remedying this gap is the explicit rationale for publicly funded efforts such as the Bridge2AI-Voice Consortium and the VOCAL consensus initiative (34). Asymmetric data sourcing from low- and middle-income countries, too, is described in a literature largely concerned with academic and Global North-led research (9, 26). What distinguishes startups is therefore not the existence of these challenges but how commercial arrangements inflect them. Where academic teams comply to mandatory institutional review, startups could elect whether to seek ethical oversight at all (theme 2), and several had not. Where academic funders and journals increasingly mandate data sharing, startups face negative incentives to share, because datasets are their principal corporate asset (theme 6). And where academic timescales at least in principle permit community engagement, investor timelines compress consent procedures and sideline participatory input (themes 3 and 5). In Leonelli's terms, the epistemic economy of voice data is field-wide, but startups occupy the position within it where commercial incentives operate with the fewest countervailing institutional checks (11). Our findings characterize how vulnerabilities across the field of vocal biomarkers are amplified in the commercial realm, rather than as identifying issues unique to startups.

The six themes identified across our interview, namely, recording protocol heterogeneity, IRB and regulatory variability, ethically ambiguous practice in informed consent, privacy issues around voice, global data extraction patterns, and commercialization tensions, are not independent problems. Rather, they are intertwined features of what we analyze below as an epistemic economy, in the sense developed by Sabina Leonelli: a system in which the social, economic, and geopolitical conditions of data production shape what counts as valid science (11).

The urgency of intervention to improve vocal biomarker research and development can be understood through Collingridge's “dilemma of control”. Early in a technology's development, its trajectory is still malleable but its effects are uncertain, whereas once it is established, its effects become clear but it grows resistant to change (29). Voice biomarker technology sits at the early, still-malleable stage. The field's own actors recognize this, as illustrated by the recent international consensus effort to standardize vocal biomarker terminology, which explicitly frames the field as being in its formative years and argues that shared standards must be established now, before proprietary systems and divergent practices become locked in (34). Our findings identify precisely the practices, unstandardized data collection protocols, opaque datasets, and asymmetric sourcing, that will be difficult to reverse once the field matures. This is the window in which governance can still shape the technology rather than merely react to it.

Leonelli, analyzing transnational plant data infrastructures, defines an epistemic economy as a knowledge system that binds together a vision for how research should be conducted, the technical tools that realize that vision, and its social and economic functions. The vocal biomarker startup ecosystem exhibits the same structure. Startups articulate a vision of universal, non-invasive health benefit. They build proprietary pipelines and datasets as the tools realizing it, and these are oriented toward commercial returns concentrated in high-income markets. Leonelli names the extractive dimension of such systems directly, describing how richer stakeholders profit from the systematic exploitation of resources held by lower-income contributors, a dynamic she terms digital bioprospecting (11). Our findings document a similar dynamic with voice data: startups described sourcing recordings from low- and middle-income countries, where compensation is lower, while orienting products toward high-income markets. We extend Leonelli's framework by characterizing this specific asymmetry in voice data as a form of epistemic exploitation: the intellectual and commercial value generated flows away from the communities whose data made it possible (31).

Beyond its ethical dimension, this asymmetric sourcing raises a distinct scientific concern. Vocal biomarkers are derived from acoustic and linguistic features of speech, yet the extent to which these features are shaped by language, dialect, and accent remains poorly characterized. A model trained on voices collected in one linguistic and cultural context may not transfer validly to a different target population, and the field currently lacks the evidence needed to know when such transfer is warranted. In the broader digital health literature, AI tools have been shown to degrade when deployed in populations that differ from their training data. A related concern has already surfaced in the vocal biomarker literature, with COVID-19 cough-screening tools showing degraded performance across populations, likely due to training data skewed toward wealthier regions (35, 36). When startups collect voice data abroad to build tools intended for use elsewhere, the resulting mismatch between source and target populations may therefore undermine not only equity but the scientific validity of the products themselves. The asymmetry we document is thus doubly consequential: it is inequitable in who bears the burden of data contribution, and it is scientifically precarious in assuming that vocal biomarkers travel unchanged across the linguistic and cultural boundaries from which they were extracted.

This problem of cross-population validity is a problem of whose knowledge the models encode. Kraft and Soulier argue that to build truly trustworthy, less biased models, we must rethink AI knowledge design (32). This involves embracing situated knowledge, acknowledging a diversity of viewpoints and cultural contexts, diversifying knowledge sources, including scrutiny from out-group members, and investing in epistemically just knowledge practices rather than defaulting to Western-centric “universal” knowledge assumptions.

The same lens applies to voice data. Although the field is young, its data collection already shows structural flaws with asymmetric sourcing, an absence of regulatory guidelines, and a lack of participatory governance. Leonelli's work suggests that addressing these dynamics requires not just better regulation but the construction of mediating institutions that can hold together the competing values, perspectives, and interests that converge in the global circulation of data. She argues that to reconcile standardization differences, there needs to be social negotiation of safe and legitimate measurement practices and ethical standards. In her work, this means involving the affected, vulnerable populations in the development of methodological practices, a practice that can be incorporated into voice AI data collection. Leonelli's account of the Crop Ontology, a “data broker” that standardized plant data across international and local stakeholders, offers a helpful alternative. Rather than imposing a single top-down standard, it developed standards in dialogue with local experts, preserving the diversity of measurement methods. This offers a concrete model for how standardization and participation can coexist, rather than trade off against each other (11).

Voice data can be used as both an important innovative tool, and work towards epistemic fairness. In his book, “The Epistemology of Resistance: Gender and Racial Oppression, Epistemic Injustice, and the Social Imagination,” José Medina argues that these knowledge injustices call for epistemic resistance, which he defines as “the use of our epistemic resources and abilities to undermine and change oppressive normative structures and the complacent cognitive-affective functioning that sustains those structures” (33). Equitable and just research therefore requires the field to scrutinize its methodological practices and to build standards that develop the field ethically rather than merely rapidly. These critiques need not imply that vocal biomarkers require wholly new ethical frameworks. Medina adds the requirement that this adaptation be explicitly oriented toward equity, rather than treated as a purely technical correction. Consistent with a contextualist over an exceptionalist approach (35), our findings suggest that existing frameworks for provenance, consent, and privacy are not absent but poorly fitted to voice data and should be adapted rather than discarded.

Fragility in scientific and ethical soundness can undermine the therapeutic relevance of vocal biomarker products, with downstream consequences for clinical decisions and patient care (26). Our findings point to concrete steps, which we organize by the actor best positioned to take them. For startups, we recommend three practices, each already demonstrated by at least one company in our sample and therefore commercially feasible. First, record and report minimum provenance metadata at the point of collection by documenting device and microphone, sampling rate and encoding, recording environment, elicitation task, and the procedure by which clinical labels were assigned, so that datasets can be compared, pooled, and audited (theme 1). Second, adopt a minimum consent standard for vocal data. The consent should be written, offer opt-in consent with a plain-language explanation of re-identifiability of vocal data and provisions for withdrawal (themes 3 and 4). Third, obtain independent ethics review for all human vocal data collection, including by private companies with no academic affiliation (theme 2). For the field collectively, we recommend adopting the emerging VOCAL consensus definitions (34) and extending them toward a shared minimum metadata standard, and building pre-competitive data infrastructure, such as the data trusts proposed by Chan et al. (27) or federated validation arrangements that permit independent verification of model claims without requiring full public disclosure (theme 6). For regulators and funders, we recommend reassessing the risk classification of voice under HIPAA- and GDPR-style frameworks to reflect its non-anonymizability (7, 8) and attaching benefit-sharing and community-engagement expectations to research and products built on data sourced from low- and middle-income settings (theme 5). We also encourage regulators to require data-disposition plans for biometric datasets, so that the fate of voice recordings is defined before, not after, a company ceases operations. Most of these recommendations have direct precedents in the governance of blood specimens (22).

The case for participatory governance is normative and is also supported by the field's own stakeholders. A survey of vocal biomarker experts, clinicians, patients, and policymakers found that participants prioritized privacy and informed consent, were unable to converge on a shared definition of ethically sourced data and repeatedly called for early involvement of affected communities in shaping data practices (30). The absence of patient and community advocacy from study design that we observed in our interviews is thus a gap the field itself recognizes but has not closed. Engaging communities in governance, including benefit-sharing arrangements for low- and middle-income contributors, would help realign the epistemic economy of voice data toward the equity its universalist rhetoric promises.

Finally, the proprietary control of data we documented has consequences beyond any single company. The VOCAL consensus initiative identifies the combination of proprietary algorithms and closed audio datasets as a black box problem that limits reproducibility and erodes trust among researchers, clinicians, and regulators (34). Leonelli's analysis anticipates where this leads with her analysis that open, transnational data systems can either resist enclosure or be coupled with restrictive intellectual property that builds new monopolies grounded in control over information rather than material resources. Our findings place voice biomarker startups at exactly this fork. Whether the field develops shared, auditable data infrastructure or consolidates into proprietary silos will determine whether independent validation, and therefore scientific robustness, remains possible.

Two further features of this ecosystem surfaced in our interviews and warrant note as directions for future research. The first concerns the fate of voice data when a startup ceases to exist. One interviewed company had halted operations after an unsuccessful fundraising round (“We were in the middle of fundraising and couldn't close the round… so we had to stop”), at which point it held psychiatric voice recordings collected under consent instruments that presupposed a continuing custodian. No interviewee described a plan for data disposition upon dissolution. Given the failure rate of early-stage companies, non-anonymizable biometric data outliving its custodian is a structural feature of this epistemic economy rather than a rare case, and governance established now must anticipate not only how startups steward data but what becomes of the data when they fail. The second is infrastructural dependence. Several startups operate on subsidized cloud credits from large technology companies, and globally collected recordings may be stored far from their contributors. Voice data contributed in LMICs thus comes to reside on commercial infrastructure controlled by, and physically located in, the Global North, a dependency that compounds the sourcing asymmetry of Theme 5 and that Leonelli's analysis would predict becomes a site of control in its own right (11).

This study has several limitations. First, our sample of eleven startups characterizes patterns present among these companies; it cannot establish the prevalence of these practices among startups across the vocal biomarker field, and our findings may not be generalizable. Second, there is potential for selection bias: recruitment through the Bridge2AI-Voice Consortium's symposium networking list likely favored companies already engaged with the academic and ethics community, that is, those most likely to have considered IRB review, consent, and data provenance. The broader startup ecosystem in vocal biomarkers may exhibit weaker practices than those we document. Third, eleven of the 27 startups we contacted agreed to participate (41%) and we were unable to systematically characterize non-responders, and companies that declined may differ from participants in their data governance practices. Fourth, interviews captured practices as described by company representatives rather than as independently verified, and social desirability may have encouraged favorable self-presentation. Finally, several authors are affiliated with the Bridge2AI-Voice Consortium, which seeks to establish data standards in this field. This position afforded access and domain expertise but may have shaped both what representatives disclosed and how we interpreted their accounts.

## Conclusion

When startups aggregate voice data collected without shared standards, across heterogeneous devices, populations, and uncontrolled conditions, the resulting noise and confounding threaten the scientific validity of any biomarker derived from them. In low-resource settings, where regulatory oversight and privacy protections are often minimal or poorly enforced, the collection of voice data by startups raises significant concerns, especially given the sensitivity of voice as personally identifiable information. Vocal biomarker research continues to collect data on a large scale, and the need for research norms becomes increasingly important in order to maintain epistemic fairness and a robust epistemic economy.

## Data Availability

The data supporting the conclusions of this article are semi-structured interview transcripts from representatives of commercial vocal biomarker startups, collected under an assurance of company and participant confidentiality. Because the vocal biomarker startup field is small and the transcripts contain commercially sensitive information about identifiable companies, the transcripts cannot be publicly released, even in de-identified form, without risking re-identification of participants. Reasonable requests concerning the data or analysis may be directed to the corresponding author and will be considered subject to the confidentiality commitments made to participants.

## Group members of Bridge2AI-Voice Consortium

Yael E. Bensoussan, Olivier Elemento, Jean-Christophe Bélisle-Pipon, David Dorr, Satrajit Ghosh, Alistair Johnson, Phillip Payne, Maria E. Powell, Anaïs Rameau, Vardit Ravitsky, Alexandros Sigaras.

## Appendix 1

**Table:**

Theme	Questions
Background questions	What was your motivation for researching voice biomarkers in relation to health? Are you currently still recruiting and/or collecting data for this database? Potential follow up: What led to the end of the study? What were some difficulties you encountered (anticipated or unanticipated)? What were some parts of data collection that were easier than expected?
Protocol questions	Did you submit an IRB to your respective institution? If so, were there difficulties or was it easier than anticipated? Can you share your consent process? Potential follow up: Did you have any difficulties and/or improvements? Describe the recording environment, microphone type, sampling rate, gain, and calibration? Can you describe your protocol from recruitment, screening, data collection, to data storage? Potential follow up: Was the patient population a convenience sample? What were the demographics? Did you collaborate with bio-acousticians, clinicians, community leaders, and/or patient advocacy groups? If so, what was their input on your project? Did you try to improve the protocol? If not, how would you have improved it?
Financial questions	Were the participants compensated? If so, what? Did you obtain a license for the dataset and did any commercial products result? How do you pay for cloud storage?
Ethical questions	What do you think are the ethical considerations with voice data collection, especially concerning the consent process? What concerns do you have regarding privacy and security of voice data related to health? By geographical region, where were the participants located? Where were researchers located? What role do you see patient advocacy groups and individuals with disorders playing in influencing the ethical and technical aspects of voice data collection?
Closing	What was the most difficult part about the project? Did you stop data collection before that was anticipated? If so, why? Can you reflect on any personal experiences or observations related to voice data collection that have left a lasting impression on you? If you could suggest one key improvement or innovation in voice data collection for disorders, what would it be and why? Is there anything else you would like to add that we did not discuss today?
